# Visual decision aids to support communication and shared decision-making: How are they valued and used in practice?

**DOI:** 10.1371/journal.pone.0314732

**Published:** 2024-12-03

**Authors:** Janneke Noordman, Désanne Noordam, Jorien van Treeck, Karen Prantl, Patricia Pennings, Petra Borsje, Maud Heinen, Yvette Emond, Ester Rake, Gudule Boland, Sandra van Dulmen

**Affiliations:** 1 Nivel (Netherlands Institute for Health Services Research), Utrecht, The Netherlands; 2 Department of Primary and Community Care, Radboud Institute for Health Sciences, Radboud University Medical Center, Nijmegen, The Netherlands; 3 Pharos, Dutch Centre of Expertise on Health Disparities, Utrecht, The Netherlands; 4 Dutch Kidney Patients Association (NVN), Bussum, The Netherlands; 5 National Association ReumaZorg Nederland (RZN), Nijmegen, The Netherlands; 6 Department of IQ Health, Radboud Institute for Health Sciences, Radboud University Medical Center, Nijmegen, The Netherlands; 7 Knowledge Institute of the Dutch Association of Medical Specialists, Utrecht, The Netherlands; Universidade Estadual do Sudoeste da Bahia - Campus de Jequié: Universidade Estadual do Sudoeste da Bahia - Campus de Jequie, BRAZIL

## Abstract

**Background:**

It is unknown how visual decision aids support communication and shared decision-making in everyday clinical practice, and how they are perceived by patients with varying levels of health literacy and their healthcare providers. Recently, three visual decision aids have been developed for renal replacement treatment, osteoarthritis of the knee, and osteoarthritis of the hip. This study aims to explore how patients and healthcare providers use and value these visual decision aids.

**Methods:**

The evaluation of the visual decision aids was performed by coding video-recorded outpatient consultations (n = 35), by conducting reflective practice interviews with healthcare providers (n = 9), and through interviews with patients (n = 29). Consultations were coded using the 5-item OPTION instrument to measure shared decision-making, and self-developed items based on the visual decision aids and user guide.

**Results:**

Both healthcare providers and patients valued the use of the visual decision aids, especially the overview page with all treatment options. Accordingly, it was observed that most providers mainly used the overview page. However, providers in nephrology discussed the individual treatment pages more often than providers in osteoarthritis care. This study also showed that most providers were unfamiliar with the user guide for the visual decision aids.

**Conclusion:**

Visual decision aids for nephrology and osteoarthritis care seem particularly useful for patients with limited health literacy. Healthcare providers in this study mainly used the overview page of the visual decision aids. Although this is valued by both providers and patients, it is also important to discuss the individual treatment pages, including the pros and cons, with patients. This study also points to differences between outpatient clinics or departments in the use and implementation of the visual decision aids. The visual decision aids for osteoarthritis are used to a limited extent. In nephrology clinics, the visual decision aid is implemented.

## Background

Shared decision-making (SDM), which is seen as the preferred model for making decisions in healthcare, requires that patients have the knowledge and skills to actively participate in the conversation and decision-making process about their treatment [1, 2]. SDM is the process where the healthcare provider (HCP) and patient decide together which medical policy is best for the patient, taking into account all options, pros and cons, patient preferences and circumstances [1, 2]. However, previous research showed that both HCPs and patients need assistance in engaging in SDM [3–5]. For example, a study into decision-making in nephrology found that some HCPs implicitly steer patients towards a certain decision through their information provision, for instance by omitting information about a treatment, emphasizing disadvantages of one treatment in favor of another, and filling in a patient’s choice [6]. At the same time, this research demonstrated that both HCPs and patients do want to engage in SDM [7].

For patients with limited levels of health literacy (LHL), communication and SDM is even more challenging as they have difficulty understanding health information, applying this information to their situation, and asking questions about their disease [8–11]. Almost 48% of the European population and 25% of the Dutch population is considered to have LHL [12, 13]. In males, the elderly, individuals with educational attainment up to the vocational level, and those with lower socioeconomic status, there is a disproportionately high prevalence of LHL [12, 14]. Furthermore, LHL is situation-dependent and can affect everyone [15], at least temporarily. Although most LHL patients prefer to participate in decision-making, they appear to ask fewer questions and have less knowledge about health and healthcare [16–18]. In addition, HCPs do not routinely adapt their communication to the patient’s health literacy level [18, 19].

To facilitate communication and SDM in clinical practice, several decision tools have been developed, such as standalone ‘patient decision aids’ (PDAs) or ‘encounter patient decision aids’ (ePDAs) [20, 21]. Due to the large amount of complex written and numerical information about the pros and cons of a certain treatment, many of these supporting tools are not useful for LHL patients [22–24]. In contrast to the complex PDAs and ePDAs, visual decision aids (or picture conversation aids) contain images with small amounts of written information in plain language. To serve the needs and capabilities of LHL patients, a previous study suggested adding pictures to ePDAs to make them accessible to all patients [25]. A recent scoping review found that visual aids developed with people who have low-literacy demonstrated statistically significant improvements in health literacy outcomes, including reported benefits in medication compliance and comprehension [26]. Offering visual tools or decision aids in addition to face-to-face conversations with HCPs can potentially benefit SDM between LHL patients and HCPs [18].

So far, it is unknown how visual decision aids help communication and SDM in everyday clinical practice in nephrology and osteoarthritis care, and how they are perceived by patients with varying levels of health literacy and their HCPs. Recently, three visual decision aids for renal replacement treatment, osteoarthritis of the knee, and osteoarthritis of the hip were developed by patient’s organizations and professional organizations together with people with LHL in the Netherlands. This study aims to explore how patients and HCPs use and value visual decision aids as supporting tools for decision-making about treatment in nephrology and osteoarthritis care.

## Materials and methods

### Design

An exploratory mixed-methods design was used to evaluate the use of visual decision aids in practice from the perspective of patients (with sufficient and with limited health literacy) and HCPs. The evaluation was performed by coding video-recorded outpatient consultations in the respective outpatient settings (quantitative) and by conducting reflective practice interviews and individual and group interviews (qualitative). We followed the COREQ checklist [27] for reporting qualitative research (S1 Annex).

### Visual decision aids and context

Three visual decision aids were evaluated in this study: one for renal replacement treatment (in Dutch: keuzekaart-in-beeld Blijvende schade aan uw nieren); one for osteoarthritis of the knee (in Dutch: keuzekaart-in-beeld voor artrose in de knie); and one for osteoarthritis of the hip (in Dutch: keuzekaart-in-beeld voor artrose in de heup). The visual decision aids are based on clinical practice guidelines and consist of a visual overview page showing all treatment options, followed by more detailed pages about the specific treatment options with images and information in plain language about the treatment, outcomes, and possible risks. The visual decision aids were developed by patient organizations and professional associations together with people with LHL, assisted by Pharos (the Dutch center of expertise on health disparities). In addition, the Dutch Eye Association did a readability check for visually impaired people.

Before the start of the project, the visual decision aid for renal replacement treatment was already in use by HCPs and their patients in several hospitals. Some of these HCPs have also been trained previously in SDM, as part of a post-graduate workshop. The two aids for osteoarthritis of the knee and osteoarthritis of the hip were not yet being used in practice because they had only recently been developed. For this study, all three visual decision aids were used in real-life practice. In nephrology, the care for a patient (and the use of the visual decision aid) involved different HCPs (e.g., nephrologist, specialized nurse, and social worker) and the conversations were more aimed at providing information. These conversations were mainly with chronic nephrology patients for whom the decision process started before this study took place. In osteoarthritis patient care, or at least in this study in outpatient care, one (or at most two) HCPs were involved (rheumatologist, rheumatology consultant or orthopedist). In the Netherlands, osteoarthritis outpatient consultations generally include a physical examination and conversation about pain management and whether or not to have surgery.

### Procedure

The study was conducted by the Dutch Centre of Expertise on Health Disparities (Pharos), Netherlands Institute for Health Services Research (Nivel), and the Department of IQ Healthcare (Radboudumc), in cooperation with the Knowledge Institute of the Dutch Association of Medical Specialists, the National Association ReumaZorg Nederland (RZN), the Dutch Kidney Patients Association (NVN), and the Dutch Federation of Nephrology (NfN)/Dutch Internists Association (NIV). The project took place between March 1^st^ and September 1^st^ 2022. HCPs–both clinicians and specialized nurses–working in Dutch hospitals at the departments of nephrology, orthopedics, or rheumatology were recruited through the network of the patient’s organizations and professional organizations involved. After receiving both an e-mail about the study and information letters for HCPs and patients, they were invited for an online meeting with a representative from the associated patient organization (PP or KP) and the researchers (JN, DN) to discuss potential participation in the study. Patients with osteoarthritis of the knee or hip and nephrology patients were approached by the participating HCPs. Participants were selected using convenience sampling. Inclusion criteria were: adult patients with osteoarthritis of the knee or/and hip, or nephrology patients. We did not have exclusion criteria. We aimed for a total of 10 participating HCPs and 40 participating patients, equally distributed over the conditions. This sample size was chosen 1) because of the explorative nature of the study, and 2) based on a reasonable sample size within the time limit of six months. All participating HCPs and patients signed an informed consent form.

#### Video recordings

Consultations between patients and their HCPs, in which one of the three visual decision aids was used, were video-recorded. To include enough patients with limited health literacy (at least 30%, preferably 50%), we asked HCPs to record all consultations where a visual decision aid was used. After the recording, researchers interviewed the patient to establish their level of health literacy. Screening questions were used for that purpose (see ‘Interviews with patients’). All video recordings were coded by one experienced observer (DN) by means of a predefined observation protocol (S2 Annex) consisting of a.) the original English 5-item OPTION instrument [28–30] and b.) self-developed items. The 5-item OPTION is a reliable and validated instrument for measuring SDM [28–30]. The 5-item OPTION demonstrated discriminative validity, concurrent validity with the 12-item OPTION (r = 0.61 (95%CI 0.54, 0.68)) intra-rater reliability (r = 0.93 (0.83, 0.97)) and lower cognitive burden of coders compared to the 12-item OPTION [29]. The 5-item OPTION consists of five items that are coded on a 5-point Likert scale, ranging from 0 = ‘zero effort observed’ to 4 = ‘exemplary effort’. The total OPTION score is generated by converting the scores to a 0–100 scale and then calculating the average, with higher scores indicating a higher level of SDM. In addition, self-developed items were included in the observation protocol, based on the visual decision aids and its user guide: e.g., introduction and use of the visual decision aid by the HCP; checking the patient’s understanding, use of teach-back; the patient’s questions and remarks about the visual decision aid, potential understanding or misunderstanding of treatment options and outcomes; discussing follow-up (S2 Annex). The first draft of the observation protocol was developed by two researchers (JN, DN) and discussed with other researchers and partners (SvD, GB, JvT). A second draft of the observation protocol was tested by two observers (DN, MvG) on two recorded consultations before it was finalized.

#### Interviews with patients

All patients who participated in the recorded encounters were asked to participate in an interview afterwards. Patients could choose a face-to-face interview directly after the recording or an interview by phone within two weeks after the recording (mean duration: 20 minutes). The interviews were semi-structured and main topics included were use and perceived assistance provided by visual decision aids, understanding of information, communication and participation in decision-making (S3 Annex). The interview guide was developed by two researchers (DN, JN) based on literature and experience from previous research [e.g., 3, 31]. Feedback was provided on the initial versions of this interview guide by the project group members (SvD, GB, JvT, PP, KP, MH, YE, ER). Interviews were conducted by one of two researchers (DN, female, MS, MA.; MvG, female, BS.). Patients had no prior knowledge of the researchers who interviewed them.

During the interview, the patient’s level of health literacy was defined using three Dutch screening questions, based on the English screening questionnaire by Chew et al. [32]. In addition, a question about the person’s educational level was asked:

- Many people have difficulty reading hospital leaflets. How is that for you?
- Many people have difficulty filling in forms. How is that for you?
- Does anyone ever help you to fill in forms or read letters?
- Educational level: the number of years a person attended school.

A person has LHL if they answer one or more of the questions in the affirmative. Previous research showed that these brief screening questions are valid to identify individuals with inadequate health literacy, and that a single is already useful in detecting patients with inadequate health literacy [33]. For the question on education, we assumed that anyone with vocational education level 2 or below falls into the group at risk of LHL or even low literacy. A patient has sufficient health literacy if they answer no to all the questions and their education level is vocational level 3 or higher. If a patient did not participate in the interview, their level of health literacy was defined by asking the HCPs and observing the recorded consultation.

In case we were not able to include enough patients with LHL (30–50% of participants), we retained the option of conducting additional interviews with people with LHL or low literacy recruited through Pharos (JvT, female, MS).

#### Reflective practice interviews with HCPs

Before conducting the reflective practice interviews, 1 to 3 fragments from the video-recorded encounters were selected for each HCP by one of two researchers (DN, MvG). The fragments selected focused on three types of occurrences in the videos (or combinations thereof): 1) potential manifestations of misunderstandings between patient and HCP, 2) elements of SDM that were present or missing, and 3) communication about the visual decision aid during the consultation and its use (or not). The HCPs were asked to 1) watch 1 to 3 fragments from the video-recorded encounters and to recall the conversation with the patient, and 2) give their perspectives on their communication, SDM, use of the visual decision aid, and other aspects that could facilitate or hinder communication and SDM with patients, including those with LHL [31]. After the reflective practice part, additional questions were asked about the use and implementation of the visual decision aid (S4 Annex). The interview guide was developed by two researchers (JN, DN) based on literature and experience from previous research [31, 34]. Feedback was provided on initial versions of the interview guide by the project group members (SvD, GB, JvT, PP, KP, MH, YE, ER). The (reflective practice) interviews with HCPs took place one to three weeks after the initial recording (mean duration: 1 hour).

### Analyses

Nine video-recorded encounters were double coded (DN, MvG) to check for sufficient inter-rater reliability. Because of the initial low agreement (44% initial agreement), disagreements were discussed with another researcher (JN) until consensus was reached and the main observer (DN) adapted the codings when necessary. Reflective practice interviews and individual interviews were audio recorded and transcribed verbatim (DN, JvT). Individual interviews with patients after the video recording, both in-person and by phone, were immediately transcribed by the researchers (DN, MvG). Transcripts were not returned to the participants for comments. Next, all interviews were analyzed by deductive thematic analyses [35]. All transcripts were read carefully and initial codings were independently assigned by one of two researchers (DN, JvT). Discrepancies between the researchers were resolved through discussion with a third researcher (JN) and modifications to the initial categories were made when necessary. All categories and patterns that emerged during analysis are illustrated by multiple quotes that have been translated into English and edited to increase readability without losing meaning or context. In the analysis of the patient interviews, we distinguished between patients with sufficient health literacy and those where it was limited.

### Ethics approval and consent to participate

The Medical Ethical Committee of the Radboud university medical center (called “CMO Oost-Nederland”) exempted this study from formal ethical approval (CMO 2022–13489) as patients were not subject to procedures nor required to follow rules of behavior (see: https://english.ccmo.nl/). The study has been performed in accordance with the Declaration of Helsinki. Written informed consent was obtained from all participating patients and HCPs.

## Results

### Participants

**Fig 1:**
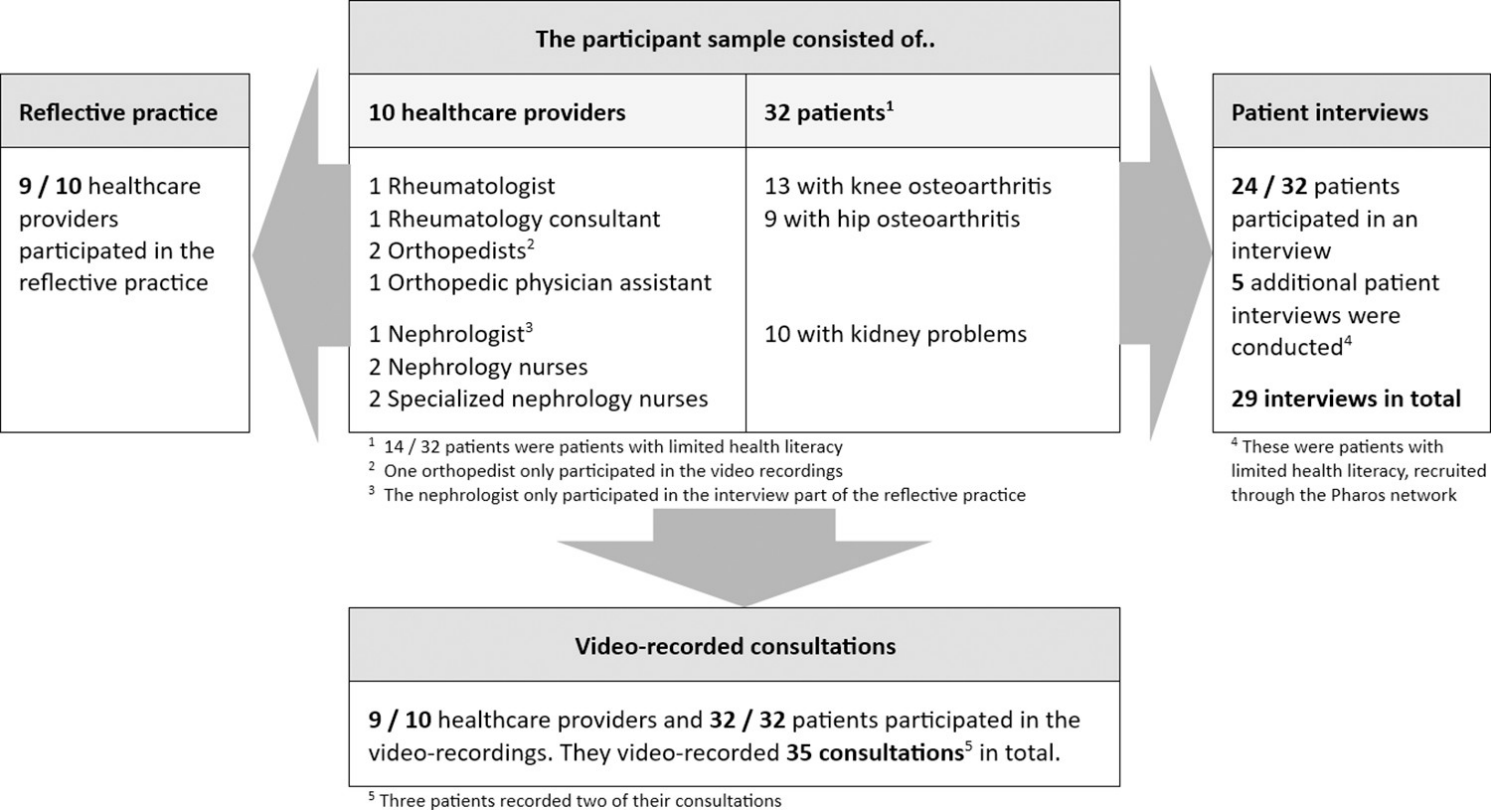
Flow chart of participants and methods used.

Fig 1 gives an overview of the participants and methods used. Ten HCPs from six hospitals and 37 patients participated in the study. Of the 32 patients who participated in the video recordings, 24 participated in the patient interviews. The health literacy levels of 8 patients who did not participate in the interviews were estimated by their HCP and by the researchers from their recording. Ultimately, twenty-three patients with sufficient health literacy (62%) and fourteen patients (38%) with limited health literacy participated. One HCP (an orthopedist) did not participate in the reflective practice and interview after the recording, while another HCP (a nephrologist) only contributed to the interview, both due to a lack of time. Sixty-six percent (n = 21) of the patients were female. The patients had an average age of 67 years (range: 46–83; n = 8 missing). Although the included percentage of participating LHL patients was in line with our aim of 30–50%, we conducted additional interviews with patients with limited health literacy or low literacy (n = 5) to gain a more in-depth understanding of their needs for SDM, communication, and the use of visual decision aids (i.e., for data saturation purposes).

The reporting of the results is structured by the data collection used as described in the method section: 1.) quantitative findings from the coded video-recorded consultations, 2.) qualitative findings from the interviews with patients and HCPs, reported in main themes (heading) and subthemes (in bold).

### 1. Findings from the coded video-recorded consultations

#### Shared decision-making in clinical practice

The observed mean shared decision-making (SDM) score was 46 (0–100 scale). This suggests that the extent to which HCPs involved patients in SDM in practice is moderate (basic phrases or sentences used). Of the OPTION scale items (see Table 1), the highest average score was observed for Item 1 (2.6; HCP drawing attention to or confirming options and the need for a decision), the lowest average score was observed for Item 2 (1.2; HCP reassures or reaffirms support to the patient for becoming informed or deliberate options). As mentioned before, the starting position for use and implementation of the visual decision aids differed substantially between HCPs working in nephrology and HCPs in osteoarthritis care (see method section). This was also reflected in the observed consultations; HCPs in nephrology showed a higher level of SDM compared to HCPs in osteoarthritis care. In addition, the duration of the consultations differed considerably, with a mean duration of 42.42 minutes for conversations in nephrology and a mean duration of 12.15 minutes for osteoarthritis care (please note: physical examinations were not recorded).

**Table 1:** Observations of recorded outpatient encounters, using the 5-item OPTION Instrument to measure the level of SDM (n = 35).

OPTION item	Mean scores*
1. For the health issue being discussed, the clinician draws attention to or confirms the fact that there are alternate treatment or management options or that a decision needs to be made *(with support of the visual decision aid*). If the patient rather than the clinician draws attention to the availability of options, the clinician responds by agreeing that the options need deliberation.	2.6
2. The clinician reassures the patient or reaffirms that they will support the patient in informing them or deliberating the options. If the patient states that they have sought or obtained information before the meeting, the clinician supports the deliberation process.	1.2
3. The clinician gives information (*or checks understanding)* about the options that are considered reasonable (this can include taking no action), to support the patient in comparing alternatives *(with support of the visual decision aid*). If the patient requests clarification, the clinician supports the process.	2.0
4. The clinician makes an effort to elicit the patient’s preferences in response to the options that have been described. When the patient states their preference, the clinician is supportive.	2.1
5. The clinician makes an effort to integrate the patient’s elicited preferences as decisions are made. If the patient indicates how best to integrate their preferences as decisions are made, the clinician makes an effort to do so.	1.7
**Total 0–100 converted score**	46 (range: 20–95)

*Possible scores: 0 = No effort (zero effort observed in the video-recorded consultation); 1 = Minimal effort (effort to communicate could be implied or interpreted in the video-recorded consultation); 2 = Moderate effort (basic phrases or sentences used in the video-recorded consultation); 3 = Skilled effort (substantive phrases or sentences used in the video-recorded consultation); 4 = Exemplary effort (clear, accurate communication methods used in the video-recorded consultation).

### Visual decision aids use in clinical practice

The observed use of the visual decision aids in clinical practice is summarized in Table 2.

**Table 2:** Observations of recorded outpatient encounters, using self-developed items to measure the use of visual decision aids in practice (n = 35).

*The use of the visual decision aids during encounters*	Scores (n = 35)
1.The healthcare provider introduces the visual decision aid by placing it visibly on the table in front of the patient and draws attention to it	Not observed: 11 Visible on the table: 4 Visible on the table and draws attention to it: 20
2. The healthcare provider explains to the patient how the visual decision aid will be used during the conversation	Not observed: 24 Observed: 11
3. The healthcare provider starts with the overview page and indicates that there are a number of treatment options that will be discussed one by one	Not observed: 9 Starts with overview page: 0 Starts with overview page and mentions treatment options: 26
4. The healthcare provider checks the boxes of the possible treatment options on the overview page and explains to the patient why some boxes are checked and others not	Not observed: 5 Provider checks the boxes: 0 Provider does not check the boxes, does give an explanation: 27 Provider checks the boxes and gives an explanation: 3
5. The healthcare provider discusses the specific treatment options on the visual decision aid that are relevant for the patient	Not observed: 25 Observed: 10
6. The healthcare provider draws attention to the red boldfase (difficult) words on the visual decision aid, discusses those words and asks if the patient understands them	Not observed: 33 By means of teach-back: 1 With some words: 2 With all words: 0
7. While discussing the treatment options, the healthcare provider asks the patient to tell them in their own words what has just been discussed (teach-back)	Not observed: 32 Observed: 3
8. The healthcare provider uses additional tools (e.g. website, pictures, powerpoint presentation) to check and/or increase the patient’s understanding	Not observed: 8 Observed: 27
9. The patient asks the healthcare provider questions (e.g. about or in relation to the visual decision aid; questions that show understanding (or not) of the treatment options and/or outcomes)	Total number: 169 Mean: 4.8 questions per conversation Range: 0–20 questions
10. The patient makes statements (other than asking questions) that demonstrate a lack of understanding/comprehension (e.g., remaining silent for a long time after an explanation from the provider, answering in a desperate tone, non-verbal questioning look*)	Not observed: 33 Observed: 2
11. When all treatment options have been discussed, the healthcare provider places the various treatment options (*i*.e., separate treatment pages of the aid) next to each other; the provider ensures that the same numbering is visible, so that the options are clear for the patient.	Not observed: 34 Observed: 1
12. The healthcare provider discusses the following steps that are necessary and, if necessary, immediately makes a follow-up appointment with the patient in which the final choice of treatment will be discussed	Not observed: 1 Observed: 34
13. After the conversation, the healthcare provider gives the visual decision aid to the patient to take home	Not observed: 5 Observed: 30

*is often not visible on the recordings, can be deduced from the healthcare provider’s comments.

It was observed that most HCPs mainly used the overview page of the visual decision aids with a single illustration of each treatment option during their consultation. HCPs also introduced the aids by means of the overview page (e.g., ‘this overview page contains all treatment options for osteoarthritis of the knee’). The separate treatment pages were used to a lesser extent. However, HCPs in nephrology discussed the separate treatment pages more often than HCPs in osteoarthritis care. HCPs did not explain to the patients why they used the aid during the consultation. A couple of HCPs did not discuss the visual decision aid during the consultation, but gave the aid to the patient to take home. Also, we observed that some HCPs communicated about the disease itself, as part of their own information provision, before discussing the visual decision aid.

Furthermore, the observations revealed that all treatment options were mentioned by HCPs when using the overview page of the visual decision aid. Two examples from the video-recordings show that this can assist SDM: 1) a rheumatologist believed that adapting the lifestyle was no longer an option for a patient but when discussing all the treatment options with the visual decision aid, the patient saw an opportunity to further adapt lifestyle behavior (no. A03-05-04); 2) An older patient decided that she did not want dialysis or a transplantation to treat her renal failure, and chose the conservative option. During the encounter, however, there was some doubt whether abdominal lavage (peritoneal dialysis) at home was still possible. At that moment the HCP decided to show the overview page of the visual decision aid to the patient and her daughter, with the advice to think about this option (no. N01-02-01).

In the recorded consultations it was observed that some patients asked questions about or related to the visual decision aids. The overall number of questions differed per patient; some asked a few questions, while others asked twenty questions (see also Table 3). For some patients, a family member or friend asked almost all questions. In nephrology, the questions were mainly practical (e.g., ‘How many dialysis centers are there and where should I go?’). In osteoarthritis care, patients’ questions were mainly disease-related (e.g., ‘Can my cartilage grow back?’) and some patients asked questions about treatment or outcomes of the treatment (e.g., ‘Does my osteoarthritis get worse with a lot of exercise?’, ‘For how long does such an injection work?’). The observations revealed that some HCPs were better at matching the level of information to patients than others. For example, it was observed that HCPs in osteoarthritis care explained the disease in plain language using X-ray images. Furthermore, we noticed that only three teach-back questions were asked to check the patient’s understanding.

**Table 3:** Quotes of interviewed patients and HCPs about the use of visual decision aids.

The visual decision aids		
Quote number	Quote	Participant number
**Overall design and content**		
1	“It [the visual decision aid] is clear. Images speak more to me than text.”	Patient (A02-04-02)
2	“No difficult words and you see step by step what to do.”	Patient (HL R1)
**Use of the visual decision aids and communication in consultations**		
3	“The overview page was used mostly. The doctor pointed out the options on it each time. The pages behind it were not used.”	Patient (A06 10 01)
4	“I show the visual decision aid after explaining the clinical picture. That’s to supplement my own informational materials, both during the encounter and to give along with the patient.”	HCP (A03-06-01)
5	“The overview page is on the table, and I point out different treatment options. I give the aid to the patient to review at home.”	HCP (A06-10-01)
6	“It is helpful (..), it is guiding, a kind of agenda. Then everything is discussed.”	Patient (A06 10 05; patient)
7	“I first asked him what he expected and then I said, what do you already know? Has [name HCP] talked to you about this [dialysis] yet? Well actually not yet. So, I first gave the initiative to the patient, I remember that very well. And [I asked] what he wanted to talk about. And then he said, well, I don’t know anything about dialysis. Then I took the visual decision aid and we went through the options [i.e., also explaining dialysis].”	HCP (N05 08)
8	“What I don’t do (..) is asking them [patients] to summarize in their own words. That does then let you determine whether they understood it. But I also think it’s kind of infantile, scholastic: adults want to be taken seriously. So, you have to think carefully about how you frame it.”	HCP (N04 07 01)
**Use of the visual decision aids and shared decision-making**		
9	“I liked the way the decision was made. The healthcare provider presented me with the options and together we made a decision. I’m a layperson and I trust the judgement of my specialist. After all, they are a specialist.”	Patient (A02 04 02)
10	“I prefer to have the final word myself, when a decision has to be made. Although, I do listen to the advice of my healthcare provider.”	Patient (A03 05 03)
11	“That’s how I know. I can see it. When he tells me [healthcare provider], I often don’t understand him. When he shows me this [the visual decision aid], I see medicines, an injection in the knee. Here they show an operation. With talking and [seeing] pictures I understand it better.”	Patient (R2)
12	“The four options are clearly explained in the visual decision aid. I find the verbal explanation from the healthcare provider the most important, but such an aid is a nice addition to the information dense consultations with the healthcare provider.”	Patient (N01 01 02)
**Patients’ characteristics and context**		
13	“I feel that the aid did not really help me. I know a lot, because my other knee has already been operated on once. If this had not been the case, I would have found the aid useful.”	Patient (A02 04 02)
14	“The visuals are pleasant. Especially when providing information to people with limited health literacy. In the second conversation they [patients] also recognized the visuals, so then the information is remembered through the visuals.”	HCP (N01-02)
15	“I do think about questions I wanted to ask [afterwards], but in 3 months I have a follow-up meeting when I can still ask [those questions]. In the meantime, the huge amount of information can sink in.”	Patient (N01 01 02)
**Implementation of the visual decision aids in hospital departments**		
16	“Every center that uses this [visual decision aid] is inventing the wheel for itself. We don’t have to do it similarly, but I did call [name other hospital] to hear how they do it. But they have their kidney outpatient clinic set up very differently to us. So it’s finding a way for yourself, how you’re going to use it [the visual decision aid].”	HCP (N04 07 01)
17	“The use of the visual decision aid is not in protocols. [We made a] mutual agreement in the department [to use the aid], but whether everyone uses it [the visual decision aid] in practice, I don’t know.	HCP (A06 10 01)
18	I am the coordinator in the renal failure working group, so I can take this [visual decision aid] to the working group and say it can be used this way and that way.”	HCP (N04 07 01)
19	“I think the family doctors might be able to start using it [the visual decision aid] a little bit more. Because actually, of course, that’s where most of the [patients’] treatment starts. The family doctor is very good at diagnosing osteoarthritis or taking a photo. (..) That saves a lot of referrals to us. They [patients] should really only come to us when they really need an operation.”	HCP (A06 09 02)

### 2. Findings from the interviews with patients and HCPs

#### The visual decision aids

*Overall design and content*. Overall, patients appreciated the design and content of the visual decision aids, especially the images (Quote 1) and the plain language (Quote 2). See Table 3 for all quotes.

Some patients with sufficient health literacy found the design too simple. Patients with a higher degree of education and more medical background were also more critical about the visual decision aids. They preferred more background information. On the other hand, some LHL patients still found the text on the pages difficult (e.g., the word ‘cartilage’). Other patients mentioned specific areas for improvement: fewer pages and more inclusive, less old-fashioned images and more space between the lines of the text. Several patients and HCPs expressed the need for additional supporting information through videos or animations (e.g., on how abdominal lavage works) and PowerPoints (i.e., to show the visual decision aid on screen during the consultation).

*Use of the visual decision aids and communication in consultations*. According to several nephrology patients, both the overview page and the separate treatment pages were discussed. Patients with osteoarthritis in particular stated that the aid was only discussed to a limited extent, i.e. mainly the **overview page** (Quote 3). As a result, osteoarthritis patients said that they did not have a strong opinion about the use of the visual decision aid. However, the additional interviews with LHL patients showed that it is important that the HCP reads through the visual decision aid together with the patient because that provides the opportunity to ask for clarification. The visual decision aid should not be handed out as an information leaflet afterwards without discussion. Some LHL patients also mentioned the importance of **introducing** the aid, to make clear that the patient has a choice and that the HCP is going to make a decision together with the patient.

Many HCPs also said they mainly used the **overview page** of the aid, and they gave the aid to the patient to take home (Quotes 4,5). In addition, several HCPs stated that they used the visual decision aids to complement their own information provided, while others said they do not use the aids during the consultation because they already provided this information without an aid as part of their daily practice. Patients’ **taking the aid home** was considered helpful by almost all HCPs. Most HCPs were not familiar with **the user guide** for the visual decision aids.

HCPs felt that the visual decision aids, especially the overview page, helped them give **structure** to the conversation and it also ensured they mention all options. Patients also perceived the visual decision aid as supportive because it gives them a better **overview and** provided **structure** during the conversation with the HCP (Quote 6).

Most HCPs said that they tried to **adapt the information** to the patients’ health literacy level, for example, by breaking up the information into chunks, avoiding difficult words and using more visuals. Other HCPs indicated that they **ask questions** to match the patients’ health literacy level. For example, by asking how someone is feeling or if someone is stressed; and by asking the patient what he or she already knows about the disease and treatment (Quote 7).

Several HCPs mentioned that they are familiar with **teach-back techniques** to check whether their explanation was understandable enough, but they all experience barriers using it (Quote 8). While patients, both with sufficient and limited health literacy, perceived this method as useful, as long as an HCP asks respectfully. During the reflective practice interview the HCP that asked a teach-back question at the end of the consultation, said that it is better to use teach-back techniques several times during the consultation, to adapt one’s communication if necessary.

Few patients reported that the visual decision aid **helped** them **to ask questions**. However, we did not specifically ask patients about this during the interviews.

*Use of the visual decision aids and shared decision-making*. Generally speaking, patients have different needs and expectations regarding SDM. Some patients particularly valued the advice of the HCP and expected the HCP to make the final decision (Quote 9). For them, the conversation with the HCP is most supportive. Other patients preferred to share the decision-making or wanted to have the final word in making the decision (Quote 10).

Many patients stated that the visual decision aids helped their **understanding of the treatment options** (Quote 11). Patients did note, however, that the aids should always **complement the face-to-face conversation** with the HCP (Quote 12).

HCPs also considered these aids helpful for patients to provide insight into their treatment options, mostly because of the visuals. However, according to HCPs, the aids also give patients a picture of **treatments that do not apply** to them, which could give the false impression that this is possible.

Although patients mentioned better understanding of the treatment options, they also said that the visual decision aids did not help their **knowledge about the pros and cons of the treatment** or **knowledge about the disease** itself. Most patients stated that this is because the HCP mainly used the overview page and not the specific pages for each treatment (with the pros and cons). Other patients were already aware of the pros and cons of treatment and were familiar with their disease.

### Patients’ characteristics and context

Patients who already **had considerable knowledge** about treatment options said that the visual decision aids assisted them less. HCPs also said this. Patients with kidney failure in particular had already been well-informed by previous information sessions or consultations. Osteoarthritis patients searched for information by themselves more often or had previous experience with osteoarthritis treatments (Quote 13).

The HCPs mentioned that the visual decision aids are **accessible for a broader group of patients** than the ‘regular’ decision aids (i.e., PDAs or ePDAs). HCPs found the visual decision aids specifically useful for LHL patients. For example, one HCP observed that LHL patients recognized the information during a follow-up consultation because of the visuals (Quote 14).

According to both patients and HCPs, the extent to which the visual decision aid is perceived as supportive also depends on **the space a patient has and feels** to receive information and look into the aid. Some patients indicated that they perceived little space because of the large amount of information, while others mentioned the tension they experience because of their condition.

The perceived support from the visual decision aid also depends on **the extent to which multiple treatment options are possible** at that moment. In some cases, a patient has only one option. For those patients, exploring the other treatment options was not perceived as helpful. In addition, patients mentioned that their **care pathway** is important, as there are often multiple information meetings or follow-up appointments to support the decision process (Quote 15). Some HCPs mentioned limited time as a barrier to the use of the visual decision aid.

### Implementation of the visual decision aids in hospital departments

The **implementation** of the visual decision aids **varies** by HCP, outpatient clinic, center or department (Quotes 16, 17).

According to the HCPs, a **clinical champion** is needed who takes the initiative to use the visual decision aid and promotes it amongst colleagues (Quote 18).

Some HCPs in osteoarthritis care suggested that the visual decision aids can also be used by colleagues outside the hospital, especially by the family doctor (Quote 19).

## Discussion

This study aimed to explore how patients and HCPs value and use visual decision aids as a supporting tool for treatment decision-making. Our study showed that both HCPs and patients valued the use of the visual decision aids, especially the overview page with all the treatment options. This page provided them with structure and overview during the conversation and ensured that HCPs mentioned all treatment options. Accordingly, it was observed that most HCPs mainly used the overview page. However, HCPs in nephrology discussed the separate treatment pages more often than HCPs in osteoarthritis care. Some HCPs did not use the visual decision aid at all during the conversation; rather, they gave it to the patient as a take-home information leaflet. In addition, HCPs did not explain why they used the aid during the consultation, while patients do prefer an introduction of the aid. This study also showed that most HCPs were not familiar with the user guide for the visual decision aids. This partly explains why HCPs did not fully use the visual decision aids as intended during their consultations. Another explanation is the different starting position for use and implementation of the visual decision aids between HCPs, as the HCPs working in nephrology had participated in a workshop about SDM and the HCPs in osteoarthritis care did not.

More specifically, we found that the visual decision aids seem particularly useful for LHL patients, as the visuals and plain text add to their knowledge about the treatment options. Another study into vulnerable populations, including those with LHL, also found that SDM requires inclusiveness in decision support materials and plain-language communication [36]. In addition, a previous study into decision-making in breast cancer suggested that a picture option grid, compared to usual care, had more impact among women with lower SES and lower health literacy [37]. A scoping review in palliative care found that offering clear health information that is understandable for LHL patients is important, but not enough. Information needs to be supplemented with other communication strategies (e.g., using short sentences, familiar words, teach-back techniques), supporting tools (e.g., visual decision aids) [38] and empathy [39, 40]. This is in line with the opinion of patients and HCPs in our study. Patients in our study also mentioned that the aids should always complement the face-to-face conversation with the HCP. This is consistent with findings from a previous focus group study into the experiences and needs of LHL patients [18]. Moreover, this study also found that an important prerequisite for SDM is that LHL patients understand their diagnosis or health problem [18]. Another study showed that, in general, patients with kidney failure first need an understanding of kidney disease to make an informed decision for a specific kidney treatment [41].

This study found a moderate level of SDM [28] in the recorded consultations. This average SDM level is comparable to that found in other settings [e.g., 30, 31]. Still, the SDM level in nephrology seems higher compared to that in osteoarthritis care (please note: this could not be tested because of the limited number of consultations). Also compared to a previous study in nephrology [6], the level of SDM in this study in the same setting was higher. As most of the HCPs in nephrology were shortly trained in SDM, whereas the HCPs in osteoarthritis care were not, this could suggests that the previous training in SDM is reflected in a higher SDM performance. In addition, the consultation duration was longer in nephrology compared to osteoarthritis care. Moreover, consultation in nephrology had a more educational purpose for patients, while consultations in osteoarthritis care include a physical examination and mainly discuss pain management and whether or not to operate. This is in line with a previous systematic review that found that both interventions to implement SDM (e.g., training in SDM) and duration of consultations are most consistently associated with higher SDM scores [30]. However, a recent systematic review showed that implementation of SDM does not necessarily lead to longer consultations [42].

It is important to note that using visual decision aids is not a goal in itself; the aids are developed to facilitate the conversation between patients and HCPs and are part of the process of SDM. In general, facilitating the process of SDM is most important, as SDM requires a change of attitude in HCPs that cannot be accomplished with a visual decision aid or other support tool only. It also demands that HCPs acknowledge the broader context of patients and discuss what is important to them [18, 41, 43, 44]. Our study also showed that patients with considerable knowledge about their disease and its treatment considered the visual decision aids as less helpful. Asking patients what they already know about their disease or treatment and/or what their treatment priority would be could be a good starting point for HCPs to adapt their communication to individual patients. However, SDM is also a shared responsibility between HCPs and patients. Patients themselves can therefore take a proactive role in the decision-making process or ask others to assist them in this process. A previous study showed that teaching SDM content to participants with LHL increased their health literacy skills for SDM. It also changed the nature of questions they would ask HCPs in a way that enabled shared health decisions [24].

### Strengths and limitations

Comparing the different perspectives and observing the actual use of visual decision aids let us obtain a realistic view of how visual decision aids can support the process of treatment decision-making. In addition, within the short duration of the project, many participants were recruited with promising outcomes for further use and implementation of the visual decision aids. Some limitations should also be mentioned. First, the starting position of the use and implementation of the visual decision aids in this study for nephrology and osteoarthritis care differed substantially. Therefore, and because of the small-scale of this study, we could not test differences in SDM or communication between these settings. Second, although we made use of a validated observation protocol to measure SDM (i.e., the 5-item OPTION [28]), additional study-specific items in the protocol were not validated. Third, SDM is a process that takes more than one consultation; the recorded consultations are a snapshot in this process. Aspects of SDM could therefore have been discussed in a previous consultation or planned to be discussed in future consultations. However, the OPTION does take previous decision-making into account when discussed during the recorded consultation. Also, we found that most HCPs were not familiar with the user guide of the visual decision aids, but we did not ask why they were not familiar with this guide. A possible explanation for HCPs’ non-familiarity could be the separate presentation of the visual decision aids (on a website for patients called thuisarts.nl) and the user guide (on the website of the FMS). Finally, the screening questions we used to measure LHL are not ideal. Potentially, we could have missed patients with a higher educational level who know little or nothing about healthcare or misclassified patients with a lower educational level as having LHL while being experts in healthcare as a result of being chronically ill.

### Implications for practice

This project resulted in several materials for HCPs aimed to enhance the use and implementation of visual decision aids for treatment decision-making. First, the visual decision aids are freely accessible for all patients and HCPs (please note that this is not a result of this project, it was already established previously) (see S5 Annex for all hyperlinks). Second, we updated the user guide (S5 Annex) for the visual decision aids, e.g., by including tips and example sentences for understandable communication and by following the steps of SDM [1, 2]. Third, a step-by-step guide (S5 Annex) for implementation of the visual decision aids at the outpatient clinic or department was written. Fourth, a short video about the correct use of the visual decision aids was developed (S5 Annex). Finally, PowerPoint sheets (S5 Annex) with the visuals and adapted text from the visual decision aids were made that could be used by HCPs in the consulting room, to complement the information they gave themselves.

For the actual use and implementation of SDM and visual decision aids, it is first of all important that HCPs achieve an adequate level of SDM and get acquainted with available supporting materials. Our study points to several facilitators for using and implementing visual decision aids: appoint a clinical champion in using these aids at every clinic or hospital department; train HCPs in SDM and use of the visual decision aids; use available instruction materials about the use of the visual decision aids. Previous research also recommends considering the role of organizational and system-level characteristics to support the implementation of SDM [45].

## Conclusions

Visual decision aids for nephrology and osteoarthritis care seem particularly useful for LHL patients. HCPs in this study mainly used the overview page of the visual decision aids. Although this is valued by both HCPs and (LHL) patients, it is also important to discuss the separate treatment pages with patients. These pages contain *inter alia* the pros and cons of the treatment options that patients need to consider before making a treatment decision. The unintended use of all or parts of the visual decision aids in this study is partly due to unfamiliarity with proper use and the user guide. This study also points to differences between outpatient clinics or departments in the use and implementation of the visual decision aids. The visual decision aids for osteoarthritis of the knee and osteoarthritis of the hip is still only used to a limited extent; participating HCPs started to use the aids during our study. In nephrology clinics, the visual decision aid for renal replacement treatment is implemented.

## Supporting information

S1 Annex COREQ checklist. (PDF)

S2 Annex Observation protocol. (DOCX)

S3 Annex Interview guide for patients. (DOCX)

S4 Annex Interview guide for healthcare providers. (DOCX)

S5 Annex Hyperlinks. (DOCX)
