# Supplementary material for: Visual decision aids to support communication and shared decision-making: How are they valued and used in practice?
Source: PLoS One. 2024 Dec 3;19(12):e0314732. doi: 10.1371/journal.pone.0314732 (PMC11614201; doi:10.1371/journal.pone.0314732)
Supplement: S2 Annex — (DOCX) [file pone.0314732.s002.docx]

**S2. Annex: Observation protocol**

**Item codes**

OPTION = 5-item OPTION [28]. * added for this study.

User guide step = steps of the user guide for the visual decision aid (see S5 for hyperlink to the user guide).

Additional = self-developed item

|  |  | **Item codes** | **Introduction to consultation and introduction to visual decision aid** |  | **Scores** |
| --- | --- | --- | --- | --- | --- |
| 1 |  | OPTION 1 | For the health issue being discussed, the clinician draws attention to or confirms the fact that there are alternate treatment or management options or that a decision needs to be made *(with support of the visual decision aid**). If the patient rather than the clinician draws attention to the availability of options, the clinician responds by agreeing that the options need deliberation. | 0.  1.  2.  3.  4. | No effort  Minimal effort  Moderate effort  Skilled effort  Exemplary effort |
| 2 |  | Additional | The healthcare provider introduces the visual decision aid by placing it visibly on the table in front of the patient and draws attention to it. | 0.  1.  2. | Not observed  Visible on the table  Visible on the table and draws attention to it. |
| 3 |  | User Guide Step 1 | The healthcare provider explains to the patient how the visual decision aid will be used during the conversation | 0.  1. | Not observed  Observed |
|  |  |  | **Discussing the visual decision aid and treatment options** |  | **Scores** |
| 4 | a | User Guide Step 2 (part)  OPTION 1 | The healthcare provider starts with the overview page and indicates that there are a number of treatment options that will be discussed one by one. | 0.  1.  2. | Not observed  Starts with overview page  Starts with overview page and mentions treatment options |
|  | b | OPTION 2 | The clinician reassures the patient or reaffirms that they will support the patient in informing them or deliberating the options. If the patient states that they have sought or obtained information before the meeting, the clinician supports the deliberation process. | 0.  1.  2.  3.  4. | No effort  Minimal effort  Moderate effort  Skilled effort  Exemplary effort |
|  | c | User Guide Step 2 (part) | The healthcare provider checks the boxes of the possible treatment options on the overview page and explains to the patient why some boxes are checked and others not. | 0.  1.1  1.2  2. | Not observed  Provider checks the boxes  Provider does not check the boxes, does give an explanation.  Provider checks the boxes and gives an explanation. |
| 5 | a | User Guide Step 2 (part) | The healthcare provider discusses the specific treatment options on the visual decision aid (options A, B, C, etc.) that are relevant for the patient. | 0.  1. | Not observed  Observed |
|  | b | OPTION 3 | The clinician gives information (*or checks understanding*)* about the options that are considered reasonable (this can include taking no action) to support the patient in comparing alternatives *(with support of the visual decision aid**). If the patient requests clarification, the clinician supports the process. | 0.  1.  2. 3. 4. | No effort  Minimal effort  Moderate effort  Skilled effort  Exemplary effort |
|  |  |  | **Checking patient’s understanding and patient’s participation** |  | **Scores** |
| 6 | a | User Guide Step 3 | The healthcare provider draws attention to the red boldface (difficult) words on the visual decision aid, discusses those words and asks if the patient understands them. | 0.  1.  2.  3. | Not observed  With some words  With all words  By means of the teach-back method |
|  |  |  |  |  |  |
| 7 | a | User Guide Step 4 | While discussing the treatment options, the healthcare provider asks the patient to tell them in their own words what has just been discussed (teach-back method). | 0.  1. | Not observed  observed |
|  | b | Additional | The healthcare provider uses additional tools to check and/or increase the patient’s understanding | 0.  1. | Not observed  Observed |
| 8 |  | Additional | The patient asks the healthcare provider questions (e.g. about or in relation to the visual decision aid; questions that show understanding (or not) of the treatment options and/or outcomes) |  | Total number (mean; range): … |
|  |  |  |  |  |  |
| 9 |  | Additional | The patient makes statements (other than asking questions) that demonstrate a lack of understanding/comprehension (e.g., remaining silent for a long time after an explanation from the provider, answering in a desperate tone, non-verbal questioning look*). *is often not visible on the recordings, can be deduced from the healthcare provider’s comments. | 0.  1. | Not observed  Observed |
|  |  |  | **Discussing patient’s preferences** |  | **Scores** |
| 10 |  | User Guide Step 5 (part) | When all treatment options have been discussed, the healthcare provider places the various treatment options (i.e., separate treatment pages of the aid) next to each other; the provider ensures that the same numbering is visible, so that the options are clear for the patient. | 0.  1. | Not observed  Observed |
| 11 | a | OPTION 4  User Guide Step 5 (part) | The clinician makes an effort to elicit the patient’s preferences in response to the options that have been described. When the patient states their preference, the clinician is supportive. | 0.  1.  2.  3.  4. | No effort  Minimal effort  Moderate effort  Skilled effort  Exemplary effort |
|  | b | OPTION 5  User Guide Step 5 (part) | The clinician makes an effort to integrate the patient’s elicited preferences as decisions are made. If the patient indicates how best to integrate their preferences as decisions are made, the clinician makes an effort to do so. | 0.  1.  2.  3.  4. | No effort  Minimal effort  Moderate effort  Skilled effort  Exemplary effort |
|  |  |  | **Closing the consultation** |  | **Scores** |
| 12 |  | User Guide Step 7 | The healthcare provider discusses the following steps that are necessary and, if necessary, immediately makes a follow-up appointment with the patient in which the final choice of treatment will be discussed. | 0.  1. | Not observed  Observed |
| 13 |  | User Guide Step 6 | After the conversation, the healthcare provider gives the visual decision aid to the patient to take home. | 0.  1. | Not observed  Observed |
