# Supplementary material for: Visual decision aids to support communication and shared decision-making: How are they valued and used in practice?
Source: PLoS One. 2024 Dec 3;19(12):e0314732. doi: 10.1371/journal.pone.0314732 (PMC11614201; doi:10.1371/journal.pone.0314732)
Supplement: S3 Annex — (DOCX) [file pone.0314732.s003.docx]

**S3. Annex: Interview guide for patients**

**Questions related to the visual decision aid**

Instructions for researcher: The researcher should first show the visual decision aid to the patient. The researcher should ask open questions. The answer options below should not be mentioned or read out loud. Please state the name of the healthcare provider that the patient just visited; do not say ‘doctor or nurse’.

1. Did [name of doctor or nurse] use this visual decision aid during your conversation?
   - Yes
   - No ( stop the interview)
2. Was it the first time you have seen this visual decision aid?
   - Yes
   - No, it was the ….. time

2a. If yes, where did you first see this visual decision aid?

2b. Did [name of doctor or nurse] gave this visual decision aid to you to take home?

1. What do you think of this visual decision aid?

(show the visual decision aid, ask questions about the content and form of the aid, for example, is it clear, readable, understandable, childish, redundant, too big etc.).

1. [Name of doctor or nurse] used this visual decision aid during your conversation. Did [name of doctor or nurse] explain why the visual decision aid was used?
   - Yes, because….
   - No
   - I don’t know/remember
2. What did you think of the information given by [name of doctor or nurse] about the visual decision aid?
   - Really clear, because….
   - Clear, because….
   - Unclear: I did not understand….
   - Really unclear: I did not understand…
   - There was no information/explanation
3. Was a treatment decision made during the conversation with [name of doctor or nurse]?

(if yes, ask who took the decision)

- - Yes, I made the decision
  - Yes, [name of doctor or nurse] made the decision
  - Yes, [name of doctor or nurse] and I made the decision together
  - No, no decision was made
  - No, a decision had been made previously
  - Other (e.g., a significant other made the decision for me), ……

1. What do you think about this?

……………………………………..

1. What do you prefer when making a decision?

……………………………..

1. Did the visual decision aid help you …(first ask the open question, then ask about the options below)
2. to know more about the disease
3. to know what treatment options I can choose from
4. to think about the pros and cons of the treatments
5. to make a decision (i.e., if a decision was made, see Question 6).
   - Very helpful
   - Helpful
   - No opinion
   - Not helpful
   - Not helpful at all
   - Not applicable (a decision had been made previously)
6. Why was (or wasn’t) it helpful?
7. Did you think anything was missing in the visual decision aid?
8. We know that a lot of people only think of questions afterwards. You just had your conversation with [name of doctor or nurse]. Is there anything you would have liked to ask [name of doctor or nurse]?
   - No
   - Yes, namely….
9. Do you have anything else you would like to tell us about the visual decision aid or its use during the conversation?

Questions about patient’s age, gender, educational level and level of health literacy (based on the Single Item Literacy Screener [32], see page 8 in the paper for questions) were also included in this interview.
