# Supplementary material for: Visual decision aids to support communication and shared decision-making: How are they valued and used in practice?
Source: PLoS One. 2024 Dec 3;19(12):e0314732. doi: 10.1371/journal.pone.0314732 (PMC11614201; doi:10.1371/journal.pone.0314732)
Supplement: S4 Annex — (DOCX) [file pone.0314732.s004.docx]

**S4. Annex: Reflective practice interview guide for healthcare providers**

General questions:

1. Is the use of the visual decision aid documented in protocols/procedures of your hospital/department or as a mutual (non-protocoled) agreement amongst colleagues? If so, how?
2. Are you aware of the existence of the user guide for the visual decision aid?

If yes, where did you get/find the user manual? If yes, what do you think of the user manual?

1. Own use of the visual decision aid:
2. Can you describe how you use the visual decision aid during your consultation?
3. How often have you worked with the visual decision aid yourself so far?
4. When (at what times) do you use the visual decision aid in your conversations? (e.g., in the first conversation or during the (number) conversation).
5. What are your experiences of using the visual decision aid so far? (positive/negative, why)
6. How comfortable do you feel using the visual decision aid? What makes you feel comfortable or uncomfortable using the visual decision aid?
7. What are you satisfied with regarding your use of the visual decision aid?
8. In which cases/situations do you feel less comfortable using the visual decision aid? What causes that?
9. What could help you in such a situation/case? (in terms of tips, conversation tools etc.).
10. Does how you use/discuss the visual decision aid vary for each patient? How?
11. Do you always try to assess your patients’ level of health literacy? How do you make that assessment/what do you base that assessment on?
12. How do you try to match the health literacy level of patients? (e.g.,  checking understanding).
13. How much does the visual decision aid help you explain treatment options to patients who have limited health literacy?
14. Do you have any other comments about the visual decision aid and/or its use in your consultation?

Reflective practice interview:

1 to 3 fragments from the video-recorded encounters were selected for each healthcare provider. The selected fragments focused on three types of occurrences (or combinations thereof) in the videos: 1) potential manifestations of misunderstandings between patient and HCP, 2) elements of SDM that were present or otherwise, and 3) communication about and (non) use of the visual decision aid during the consultation.

1. Do you remember this fragment?
2. What do you think about the way you communicated with the patient?
3. And vice versa: What do you think of the way the patient communicated with you?
4. (Depending on the fragment, in relation to the patient’s understanding). What is your assessment of the patient’s health literacy? What makes you think that? How did you follow up on this during your conversation with the patient?
5. Can you reflect on the way you used the visual decision aid in this fragment?
6. Is the way you discussed the visual decision aid in this fragment representative of how you generally do it?
7. (Depending on the fragment, in relation to Shared Decision-Making by means of the 5-item Option [28]). Can you reflect on the way you (choose what is applicable) discussed the pros and cons of the treatment/made the treatment decision/discussed what is important for the patient in making such a decision etc.?
