# Supplementary material for: Visual decision aids to support communication and shared decision-making: How are they valued and used in practice?
Source: PLoS One. 2024 Dec 3;19(12):e0314732. doi: 10.1371/journal.pone.0314732 (PMC11614201; doi:10.1371/journal.pone.0314732)
Supplement: S5 Annex — (DOCX) [file pone.0314732.s005.docx]

**S5.Annex. Hyperlinks visual decision aids (in Dutch)**

Visual decision aids (keuzekaarten-in-beeld):

[Keuzekaart | Federatie Medisch Specialisten (demedischspecialist.nl)](https://demedischspecialist.nl/themas/thema/samen-beslissen-0/keuzekaart).

[Overzicht keuzekaarten | Thuisarts.nl](https://www.thuisarts.nl/overzicht/keuzekaarten)

User guide (updated version):

[handleiding_keuzekaart_in_beeld.pdf (demedischspecialist.nl)](https://demedischspecialist.nl/sites/default/files/2022-07/handleiding_keuzekaart_in_beeld.pdf)

Step-by-step guide for implementation of the visual decision aids at the outpatient clinic or department :

[stappenplan_keuzekaart_en_keuzekaart_in_beeld.pdf (demedischspecialist.nl)](https://demedischspecialist.nl/sites/default/files/2022-11/stappenplan_keuzekaart_en_keuzekaart_in_beeld.pdf)

A short video about the correct use of the visual decision aids:

[Keuzekaart-in-beeld - Passende behandeling kiezen | Pharos, expertisecentrum gezondheidsverschillen - YouTube](https://www.youtube.com/watch?v=rHPjDernYuE)

PowerPoint sheets, to complement the education of the healthcare providers:

[Keuzekaart | Federatie Medisch Specialisten (demedischspecialist.nl)](https://demedischspecialist.nl/themas/thema/samen-beslissen-0/keuzekaart)
